# Current of injury amplitude during left bundle branch area pacing implantation: impact of filter settings, ventricular pacing, and lead type

**DOI:** 10.1093/europace/euae130

**Published:** 2024-05-16

**Authors:** Haran Burri, Valérian Valiton, Alberto Spadotto, Julia Herbert, Nicolas Masson

**Affiliations:** Cardiac Pacing Unit, Department of Cardiology, University Hospital of Geneva, rue Gabrielle Perret Gentil 4, 1211 Geneva, Switzerland; Cardiac Pacing Unit, Department of Cardiology, University Hospital of Geneva, rue Gabrielle Perret Gentil 4, 1211 Geneva, Switzerland; Cardiac Pacing Unit, Department of Cardiology, University Hospital of Geneva, rue Gabrielle Perret Gentil 4, 1211 Geneva, Switzerland; Cardiac Pacing Unit, Department of Cardiology, University Hospital of Geneva, rue Gabrielle Perret Gentil 4, 1211 Geneva, Switzerland; Cardiac Pacing Unit, Department of Cardiology, University Hospital of Geneva, rue Gabrielle Perret Gentil 4, 1211 Geneva, Switzerland

**Keywords:** Left bundle branch area pacing, Conduction system pacing, Implantation, Current of injury, Perforation, Filters

## Abstract

**Aims:**

Monitoring current of injury (COI) during left bundle branch area pacing (LBBAP) implantation is useful to evaluate lead depth. Technical aspects for recording COI amplitude have not been well studied. Our aims were to evaluate the impact of high-pass filter settings on electrogram recordings during LBBAP implantation.

**Methods and results:**

Consecutive patients with successful LBBAP implantation had unipolar recordings of COI at final lead position at different high-pass filter settings (0.01–1 Hz) from the tip electrode during sensing and pacing, and from the ring electrode during sensing. Duration of saturation-induced signal loss was also measured at each filter setting. COI amplitudes were compared between lumenless and stylet-driven leads. A total of 156 patients (96 males, aged 81.4 ± 9.6 years) were included. Higher filter settings led to significantly lower COI amplitudes. In 50 patients with COI amplitude < 10 mV, the magnitude of the drop was on average 1–1.5 mV (and up to 4 mV) between 0.05 and 0.5 Hz, meaning that cut-offs may not be used interchangeably. Saturation-induced signal loss was on average 10 s at 0.05 Hz and only 2 s with 0.5 Hz. When pacing was interrupted, the sensed COI amplitude varied (either higher or lower) by up to 4 mV, implying that it is advisable to periodically interrupt pacing to evaluate the sensed COI when reaching levels of ∼10 mV. Lead type did not impact COI amplitude.

**Conclusion:**

High-pass filters have a significant impact on electrogram characteristics at LBBAP implantation, with the 0.5 Hz settings having the most favourable profile.

## Graphical Abstract

**Graphical Abstract:**
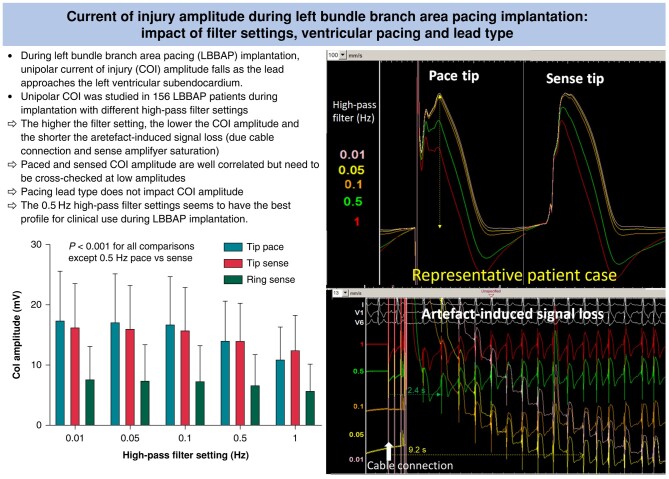

**What’s new?**

- Higher cut-off frequencies of high-pass filters result in lower unipolar current of injury (COI) values during left bundle branch area pacing (LBBAP) implantation.
- COI amplitudes during pacing and sensing are well correlated, although at values < 10 mV (i.e. when approaching the left ventricular endocardium), they should be cross-checked to evaluate agreement.
- Artefact-induced signal loss due to cable connection is significantly longer with low cut-off frequencies of high-pass filters.
- Lead type (stylet-driven or lumenless) does not impact COI amplitude.
- The 0.5 Hz high-pass filter setting seems to have the best profile for clinical use during LBBAP implantation.

## Introduction

Adoption of conduction system pacing (CSP) has risen markedly over the last years, especially as regards left bundle branch area pacing (LBBAP) as it offers physiological pacing without the limitations of His bundle pacing.^1,2^ During LBBAP implantation, the lead is advanced deep into the interventricular septum to reach the left ventricular subendocardial region where the conduction tissue lies. While striving to achieve conduction system or left ventricular septal capture, it is important to carefully monitor lead depth to avoid perforation, which may occur in up to 14% of patients.^3–6^ A number of different parameters may indicate lead depth and distance from the left ventricular subendocardium, such as paced QRS morphology, pacing impedance (which also depends on the leads and cables used), presence of a fascicular potential (which is not always visible), contrast injection and evaluation of the hinge point (which are imprecise in case of an oblique course of the lead), and unipolar current of injury (COI) amplitude/morphology.^7^

Ponnusamy *et al*.^3^ observed a drop in unipolar sensed COI from 15.4 ± 11.6 mV just before perforation, to 0.9 ± 0.6 mV after perforation into the left ventricle, with a ‘QS’ electrogram morphology, presumably due to full helix perforation. In a study by Shali *et al*.,^6^ absence of a tip COI was 100% sensitive for perforation, with tip < ring COI amplitudes having positive and negative predictive values of 57% and 100%, respectively. Based on these data and on clinical experience, a recent European Heart Rhythm Association (EHRA) consensus document on CSP implantation recommended caution against advancing the lead any further when the COI amplitude falls to 3–5 mV to avoid septal perforation.^3^

The caveats with these findings are that high-pass filter settings varied in these reports, being either 0.05 mV^6^ or 0.5 mV.^3^ We have previously shown that high-pass filter settings can cause considerable distortion of the ST-segment of surface ECGs,^8^ and it is unknown to what extent these settings may impact endocardial COI amplitude. This is also relevant in centres where pacing system analysers (PSAs) are used for monitoring COI (as these devices have high-pass filters of up to 1 Hz). Furthermore, disconnection and reconnection of pacing cables create an artefact that saturates the sense amplifier, leading to transient loss of the signal that may cause delay in the procedure. It is unknown to what extent high-pass filters impact this phenomenon. In addition, all reported cut-offs relate to COI amplitude during sensing. It is useful to be able to monitor this parameter during pacing with uninterrupted lead deployment,^9^ thereby heralding impending lead perforation and avoiding this complication. Finally, all reports relate to 4.2 F lumenless leads (LLLs), and it is unknown if stylet-drive leads (SDLs), which have larger diameters, result in different COIs.

Our aims were, during LBBAP implantation, to (1) evaluate unipolar tip and ring COI amplitudes using different high-pass filter frequencies, (2) evaluate impact of high-pass filters on duration of saturation-induced signal loss, (3) compare sensed and paced COI amplitudes, and (4) compare COI amplitude of SDLs with LLLs.

## Methods

The investigation was conducted at the University Hospital of Geneva in consecutive patients who had successful LBBAP implantation. Two physicians (H.B. and V.V.) performed the procedure using the technique described in the EHRA consensus document.^10^ The Medtronic (Minneapolis, USA) 3830 LLL as well as SDLs from three manufacturers were used (Solia, Biotronik, Berlin, Germany; Tendril STS, Abbott, Sylmar, USA; Vega, Microport, Shanghai, China). A Boston Scientific LABSYSTEM PRO electrophysiology recording system was used for implantation in all cases with continuous recording at 1000 Hz sampling rate. High-pass filters were set at 0.01, 0.05, 0.1, 0.5, and 1 Hz. A high-pass filter recording of 10 Hz was abandoned after it became apparent that the COI was almost invisible at this setting. The low-pass filter was set at 500 Hz for all intracardiac channels. An Abbott PSA was connected using Y-adapters to monitor pacing impedance and for the threshold tests. Once the target area was reached (aiming to achieve confirmed or likely conduction system capture according to current criteria,^7^ or if not possible, left ventricular septal pacing), the unipolar COI was immediately recorded during unipolar pacing from the lead tip at 5 V/0.5 ms and during unipolar sensing from the lead tip, followed by cathodal sensing from the ring (in order to avoid having a drop in COI over time). Measurements at different filter settings were made simultaneously. COI amplitude was measured directly after the procedure using the digital callipers of the recording system by one of three observers (H.B., V.V., and A.S.). Amplitude was measured from the onset of the fast upstroke of the ventricular electrogram up to the peak of the ‘dome’ of the COI (after the sharp component of the ventricular electrogram). Thus, 15 COI amplitude measurements were performed in each patient (at the five filter settings during pacing from the lead tip and sensing from the lead tip and ring). Duration of saturation-induced signal loss was measured from the artefact resulting from reconnection of the crocodile clip to the lead, up to baseline stabilization of the signal in each of the five channels with the different filter settings.

The investigation was conducted according to the Declaration of Helsinki. All patients gave written informed consent and were part of the Geneva Conduction System Registry that had been approved by the institutional Ethics Committee.

### Statistical analysis

Analyses were performed using GraphPad Prism v8 (GraphPad software, Boston, MA). Normality of data distribution was confirmed using the Kolmogorov–Smirnov test. One-way analysis of variance (ANOVA) and Student’s test were used for testing differences between groups. Pearson’s test was used to evaluate correlation. Bland–Altman analysis was used to evaluate bias and 95% limits of agreement between different testing conditions. Results were considered to be significant at *P* < 0.05.

## Results

A total of 156 patients were included (demographics are shown in *Table 1*). A LLL was implanted in 55 (35%) and an SDL in 101 (65%) patients. Paced QRS (measured from pacing spike onset) was 159 ± 22 ms. A fascicular potential was visualized in 36 (23%) patients, and QRS transition with decrementing unipolar output was observed in 43 (28%) patients. Confirmed and likely conduction system capture according to the 2023 EHRA consensus document^10^ was attributed to 84 (54%) and 36 (23%) patients, respectively, and 36 (23%) of patients were diagnosed as having left ventricular septal pacing.

**Table 1:** Patient demographics

	*n* = 156
Male	96 (62%)
Female	60 (38%)
Age (years)	81.4 ± 9.6
Comorbidities	
Diabetes	47(30%)
Hypertension	122 (78%)
Ischaemic heart disease	67 (43%)
Valve surgery	10 (6%)
TAVI	20 (13%)
Renal insufficiency	54 (33%)
Pacing indication	
AVB I/II/III	5 (3%)/22 (14%)/43 (28%)
Slow atrial fibrillation	17 (11%)
Syncope	11 (7%)
Pace and ablate	22 (14%
CRT (in lieu of coronary sinus)	15 (10%)
LOT-CRT	9 (6%)
Sick sinus syndrome	3 (2%)
Other	9 (6%)
LVEF (%)	51 ± 15
Rhythm	
Sinus	105 (67%)
Atrial fibrillation/flutter	51 (33%)
QRS morphology	
Normal	57 (37%)
LBBB	28 (18%)
RBBB	26 (17%)
RBBB + LAFB	17 (11%)
LABB	3 (2%)
NIVCD	11 (7%)
Paced rhythm	14 (9%)
QRS duration (ms)	136 ± 37
Lead implanted	
3830	82 (53%)
Solia	52 (33%)
Tendril	19 (12%)
Vega	3 (2%)

AVB, atrioventricular block; CRT, cardiac resynchronization therapy; LOT-CRT, left bundle branch optimized CRT; LAHB, left anterior fascicular block; LVEF, left ventricular ejection fraction; NIVCD, non-specific intra-ventricular conduction delay; TAVI, transcatheter aortic valve implantation.

**Figure 1:**
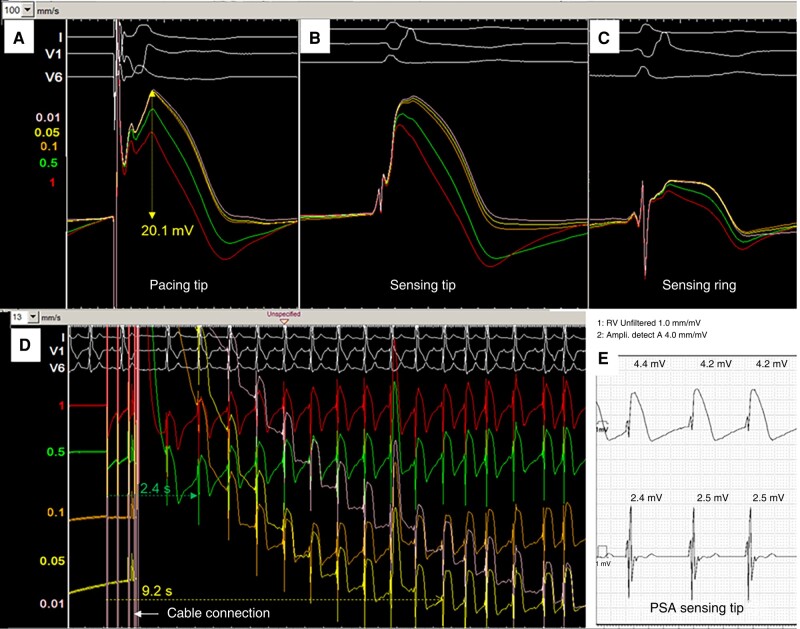
Illustrative case example in a patient during LBBAP implantation showing ventricular waveforms at different high-pass filter frequencies (values in Hz are shown in their corresponding colours). (*A*) During pacing from the lead tip, the COI amplitudes only show small differences between 0.01, 0.05, and 0.1 Hz, whereas there is a notable difference between 0.05 (20.1 mV), 0.5 (17.4 mV), and 1 Hz (13.3 mV). (*B*) During sensing from the lead tip, compared to during pacing, the COI is slightly lower at 0.01–0.05 Hz, similar at 0.05 Hz, and slightly higher at 1 Hz. (*C*) During sensing from the ring, the COI amplitudes are all lower than from the tip, with the same trends in differences between the filter settings compared to waveforms from the tip. (*D*) Measuring cable connection to the lead tip resulting in an artefact with sensing amplifier saturation and signal loss, at variable durations until baseline stabilization, depending upon filter setting (e.g. 2.4 s at 0.5 Hz and 9.2 s at 0.05 Hz). (*E*) Simultaneous recording of the waveforms via a Merlin (Abbott) PSA from the lead tip (the high-pass filter of the ventricular ‘unfiltered’ channel is 1 Hz). Note that the measured amplitude of 4.2 mV (measured via the filtered ‘Ampli-detect’ channel, which is not shown here) corresponds to the sharp deflection of the ventricular electrogram (the COI amplitude is ∼13 mV and corresponds roughly to that measured at 1 Hz via the electrophysiology recording system shown in *B*. Note also that the filtered signal (using the atrial channel simultaneously displaying filtered signals from the LBBAP lead) shows much lower sensing amplitudes due to differences in ‘Ampli-detect’ filter settings compared to the ventricular channel.

A representative example of the COI waveforms obtained with the different settings is shown in *Figure 1*.

### High-pass filter setting and current of injury amplitude

**Figure 2:**
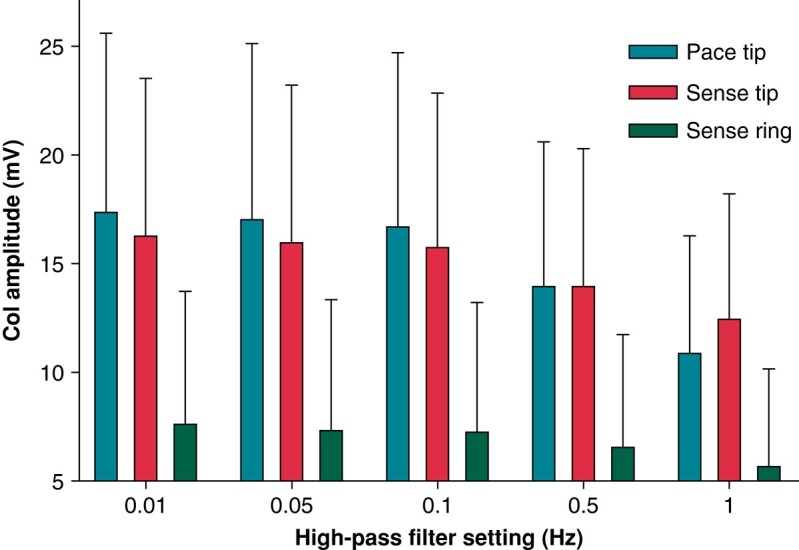
Unipolar COI at different filter settings and with pacing or sensing for the entire patient cohort (*n* = 156). All comparisons were statistically significant at *P* < 0.0001, except between pacing and sensing at 0.5 Hz (*P* = 0.88).

There was a significant fall in COI amplitude with increasing high-pass filter setting (*P* < 0.001 for all comparisons, see *Figure 2*) except for a non-significant fall in ring COI sensing amplitude between 0.01 and 0.05 Hz (*P* = 0.56). Although statistically significant, the fall in tip COI was clinically irrelevant (on average <0.5 mV) between 0.01 and 0.1 Hz, but more substantial (on average ∼2–3 mV) between 0.1 and 0.5 Hz and between 0.5 and 1 Hz.

Tip/ring COI amplitudes were consistent as being either >1 or <1 across all filter settings, although the magnitude of the ratios varied considerably and there was poor correlation between tip and ring COIs, all *r* < 0.27 (data not shown).

**Figure 3:**
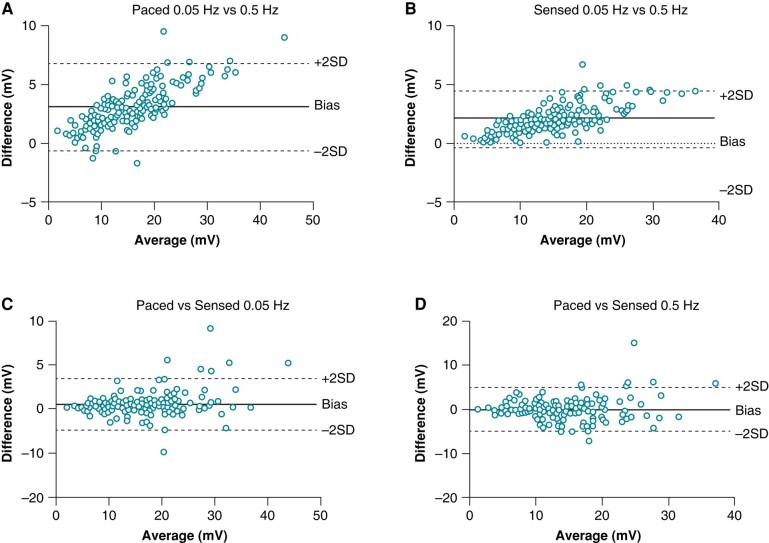
Bland–Altman plots for unipolar COI amplitudes at high-pass filter settings of 0.05 Hz vs. 0.5 Hz during ventricular pacing (*A*) and sensing (*B*), showing greater impact of filter settings at higher COI values. Comparison of COI amplitudes during pacing and sensing at 0.05 Hz (*C*) and 0.5 Hz (*D*) filter settings, with similar distribution across average amplitudes.

**Figure 4:**
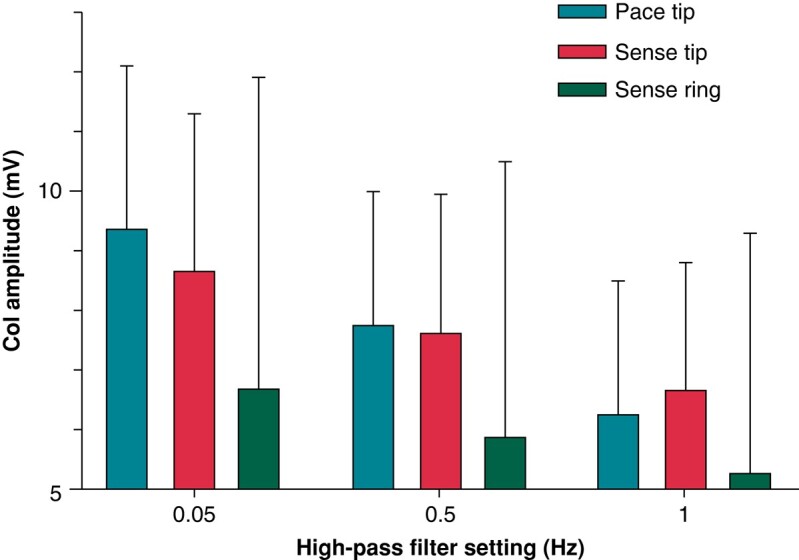
Unipolar COI at different filter settings and with pacing or sensing for the subgroup of patients with COI < 10 mV at ≤0.5 Hz settings (*n* = 51). For the sake of clarity, only data for 0.05, 0.5, and 1 Hz are shown. All comparisons between filter settings as well as between sensed tip and ring COI amplitudes were statistically significant at *P* < 0.001. There was a slight but significant fall in COI amplitude between pacing and sensing at 0.05 Hz (*P* = 0.003), with no significant differences at 0.5 Hz (*P* = 0.40), and a trend in rise at 1 Hz (*P* = 0.13).

Correlation of COI amplitudes between different filter settings was *r* > 0.98, *P* < 0.0001 for all comparisons. Bland–Altman plots clearly indicated that higher COI amplitudes were more impacted by high-pass filter settings (see *Figure 3*). Low COI amplitudes are meaningful during implantation as they indicate that the left ventricular septum is being approached. We therefore performed a subgroup analysis in patients who had a tip sensed or paced COI < 10 mV with any filter setting ≤ 0.5 Hz (the 1 Hz cut-off has never been studied previously). The data in the 51 patients who were thus identified are shown in *Figure 4*. The trends were identical to those of the overall population with the mean differences (bias) and 95% confidence intervals listed in *Table 2*.

**Table 2:** Results in the subset of patients with COI < 10 mV

Comparison	Mean difference (mV)	95% limits of agreement (mV)
High-pass filter setting		
Tip 0.05 Hz vs. 0.5 Hz		
Paced COI	1.5	−0.9–4.0
Sensed COI	1.1	−0.2–2.4
Tip 0.5 Hz vs. 1 Hz		
Paced COI	1.5	−0.3–3.3
Sensed COI	1.0	−0.1–2.0
Ring 0.05 Hz vs. 0.5 Hz		
Sensed COI	0.7	−0.9–2.2
Ring 0.5 Hz vs. 1 Hz		
Sensed COI	0.8	−0.6–2.2
Pacing vs. sensing (via tip only)		
0.05 Hz	0.6	−2.3–3.5
0.5 Hz	0.2	−2.8–3.1
1 Hz	−0.4	−4.0–3.2

Comparison by Bland–Altman analysis with mean difference (bias) and 95% limits of agreement (bias ± 2 SD) between different high-pass filter settings and between pacing and sensing from the lead tip. A positive difference means that the first setting yields higher value. The lower the filter value, the higher the measured COI amplitude (*P* < 0.001 for all comparisons).

### High-pass filter settings and duration of saturation-induced signal loss

**Figure 5:**
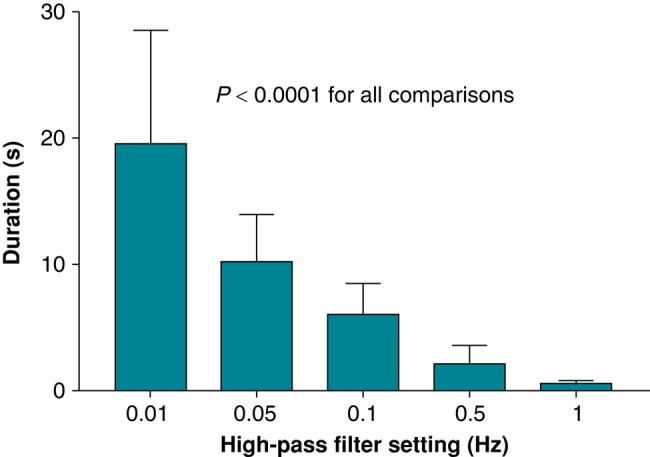
Duration of artefact-induced signal loss at different high-pass filter settings. All comparisons are significant (*P* < 0.0001). Values indicate mean ± SD.

There was a clear increase in duration of signal loss at lower filter frequencies—see *Figures 1* and *5*. The mean duration was 10.2 ± 3.7 s at 0.05 Hz compared to 2.2 ± 1.3 s at 0.5 Hz and 0.5 ± 0.2 s at 1 Hz.

### Difference in current of injury amplitude between pacing and sensing from the lead tip

For the 156 patients, with high-pass filter settings at 0.01, 0.05, and 0.1 Hz, there was a slight fall in COI amplitude when pacing was interrupted and the sensed signal was measured (*P* < 0.0001 for all comparisons). Differences were not statistically significant for filter settings of 0.5 Hz (*P* = 0.88). A slight rise in COI was noted at 1 Hz (*P* < 0.0001)—see *Figure 2*. Correlation between paced and sensed COI amplitudes was *r* > 0.92 for all filter settings except for 1 Hz where it was *r* = 0.87 (*P* < 0.0001 for all comparisons). Bland–Altman analysis did not show an impact of pacing on COI amplitude—see *Figure 3*.

In the subgroup of 51 patients with COI < 10 mV, the results were similar, but were less significant due to the smaller numbers—see *Table 2* for mean differences with 95% limits of agreement.

### Effect of lead type on current of injury amplitude

There were no differences in COI amplitudes between lead types at all filter settings. The COI amplitude at all settings for LLLs was 15.5 ± 7.1 mV and for SDLs, it was 14.6 ± 6.9 mV (*P* = 0.46).

## Discussion

Our data indicate that COI amplitudes are comparable at high-pass filter settings between 0.01 and 0.1 Hz, but fall significantly at 0.5 and 1 Hz. This means that filter settings of 0.05, 0.5, and 1 Hz may not be used interchangeably. Abbott PSAs have ‘unfiltered’ frequencies ranging from ∼1–100 Hz, whereas with Boston Scientific, they are 0.5–80 Hz and with Biotronik Renamic 0.27–200 Hz (Medtronic was unwilling to disclose its PSA filter settings). This implies that the waveforms displayed by PSAs are not equivalent amongst themselves or compared to those displayed by electrophysiology recording systems. The case example in *Figure 1* illustrates that the sensed amplitude automatically measured by the PSA may not correspond to the COI amplitude (but rather to the high-frequency component corresponding to the QRS complex), and that this value also depends on which channel the lead is connected to.

The impact of filter settings on COI amplitude in the subgroup of patients with COI <10 mV (suggesting greater proximity to the left ventricular subendocardium) is clinically relevant. For example, at 0.05 Hz compared to 0.5 Hz, COI amplitudes are higher by an average of ∼1 mV, and may differ by up to ∼5 mV. The magnitude of these differences may sway the decision to advance the lead any further. By applying the recommended sensed COI amplitude cut-off of ∼3–5 mV to stop any further advancement of the lead,^7^ it is more conservative and prudent to use a filter setting of 0.5 Hz rather than 0.05 Hz, as the former will reach those values first at a given lead depth. On the other hand, excessive filtering of COI may limit its diagnostic value, which could possibly be an issue with the 1 Hz filter setting. Tip/ring COI amplitude ratios were consistent in being either <1 or >1 across the different filter settings, and can therefore be all used for the parameter described by Shali *et al*.^6^ It is not surprising that tip and ring COIs were poorly correlated, as COI on the ring electrode will depend on its depth within the septum that may vary according to septal thickness and obliqueness of its course.

The second finding is that duration of saturation-induced signal loss is long at ∼10 s on average at 0.05 Hz filter setting, compared to only ∼2 s at 0.5 Hz and almost absent at 1 Hz. Low-frequency signals are more prone to saturation due to their longer periods, and reducing their amplitude through filtering can help prevent saturation-induced signal loss. Waiting for the signal to reappear on the screen and to stabilize causes unnecessary delay during implantation, which is yet another reason for preferring the 0.5 Hz setting to 0.05 Hz.

The third finding is that COI amplitudes are well correlated between those measured during pacing and sensing. However, at lower amplitudes (where they are most informative to indicate impending perforation), the differences may be clinically relevant (up to ∼6 mV for 0.05 and 0.5 Hz, and even ∼7 mV at 1 Hz). It is useful to continuously pace via the lead while it is being deployed to evaluate its depth by monitoring paced QRS morphology, impedance, and COI amplitude. When the paced COI reaches an amplitude of ∼10 mV, it may be advisable to transiently interrupt pacing to check the sensed COI amplitude. Gain settings may be adjusted to be able to eyeball COI amplitude on the screen (e.g. by scaling 10 mV to about a quarter of the screen height); this allows to visualize significant drops in COI amplitude while screwing the lead, and to only perform manual measurements when low amplitudes are reached. This strategy may avoid inadvertent septal perforation, and can be used in addition to the aforementioned parameters to determine when to stop advancing the lead. The 95% limits of agreement were wider for 1 Hz than for the other filter settings, meaning that when pacing is stopped, the sensed COI is less predictable at this setting.

Finally, it is not surprising that lead type (LLL vs. SDL) does not impact COI amplitude, as this is probably more related to lead depth (and proximity to the left ventricular subendocardium) than to lead diameter (and tissue trauma).

In our current daily practice with LBBAP implantation, monitoring of tip paced and sensed COI amplitudes has proven to be extremely valuable to gauge lead depth and proximity to the left ventricular subendocardium and to thereby avoid perforation. It is also valuable to identify micro-dislodgement, which is suggested by an *increase* in COI compared to that measured at the targeted lead position.

### Study limitations

It is important to note that different types of filters exist, such as Butterworth, Chebyshev, and Bessel filters. Each type has its own characteristics in terms of frequency response, phase response, and transient response, thus impacting COI waveforms in addition to high-pass filter frequency. Our study did not compare head-to-head the COI recorded by different systems (including PSAs), and our results are limited to the LABSYSTEM PRO. Due to confidentiality issues, manufacturers are reluctant to disclose technical details of their hardware, and it is difficult to ascertain to what extent our results may be extrapolated to other systems.

The effect of low-pass filters (i.e. which filter out high frequencies) was not studied, but it is unlikely that these impact COI due to its predominantly low frequency.

Visualization of fascicular potentials using different high-pass filters was not evaluated. However, these potentials are sharp (i.e. they are constituted by high-frequency components) and are rather more likely to be impacted by low-pass filter settings. The gain settings of our unfiltered electrographs were set for evaluating myocardial COI, and not fascicular COI (which are much lower in amplitude).

We did not evaluate bipolar COI signals, as these are simply the difference between the tip and ring unipolar waveforms. Thus, in the instance of ring > tip COI amplitude, the bipolar COI will be negative.

Abnormal septal substrate due to scar or amyloid disease may have affected COI amplitude, but this was not evaluated due to limited availability of imaging data. Nevertheless, we expect that high-pass filter settings, and pacing, are likely to impact waveforms in a similar manner as compared to normal tissue.

Our study is not intended to provide cut-offs of COI amplitudes for diagnosing septal perforation. This should ideally be performed by a study that visualizes lead tip position in real-time to evaluate COI amplitude at different depths (e.g. using perioperative intracardiac echocardiography).

## Conclusion

High-pass filter settings significantly impact COI amplitude (with lower amplitudes at higher frequencies), which should be borne in mind when defining cut-offs for evaluating perforation. The setting of 0.5 Hz seems to have the best overall characteristics. Monitoring of COI during continuous pacing can be performed, but it is advised to check sensed COI amplitude when amplitudes fall to ∼10 mV as there may be considerable differences between paced and sensed COI amplitude. Lead type does not impact COI amplitude. Our findings set the stage for further research to better define cut-offs for diagnosing impending or confirmed septal perforation.

## Data Availability

De-identified data will be made available upon reasonable request to the corresponding author.
